# miRNA-99b-5p suppresses liver metastasis of colorectal cancer by down-regulating mTOR

**DOI:** 10.18632/oncotarget.4423

**Published:** 2015-06-10

**Authors:** Wenhua Li, Jinjia Chang, Shanshan Wang, Xinyang Liu, Junjie Peng, Dan Huang, Menghong Sun, Zhiyu Chen, Wen Zhang, Weijian Guo, Jin Li

**Affiliations:** ^1^ Department of Medical Oncology, Fudan University Shanghai Cancer Center, Shanghai, China; ^2^ Department of Oncology, Shanghai Medical College, Fudan University, Shanghai, China; ^3^ Department of Colorectal Surgery, Fudan University Shanghai Cancer Center, Shanghai, China; ^4^ Department of Pathology, Fudan University Shanghai Cancer Center, Shanghai, China

**Keywords:** colorectal carcinoma, liver metastasis, miR-99b-5p, mTOR

## Abstract

Liver metastasis is common in patients diagnosed with colorectal cancer (CRC), and is also correlated with poor outcome. In this study we screened the different expression profiles of microRNAs (miRNAs) on the development of liver metastasis in CRC patients. miR-99b-5p was found to be more than 6-fold higher in primary tumors than in matched liver metastases (*P* = 0.007). Expression of miR-99b-5p in primary tumors of patients with stage III CRC without liver metastases was higher than in CRC patients with liver metastases (*P* = 0.028). Up-regulated miR-99b-5p was associated with longer overall survival (*P* = 0.01). Besides, miR-99b-5p silencing in miR-99b-5p-positive CRC cell lines promoted cell migration and up-regulated mTOR, and vice versa. In addition, luciferase assays demonstrated that miR-99b-5p functioned as a tumor suppressor by targeting mTOR. Taken together, our results demonstrate thatmiR-99b-5p is differently expressed in primary CRC and liver metastasis and functions as a tumor-suppressive microRNA in metastatic CRC. The miR-99b-5p–mTOR axis may serve as a prognostic factor and therapeutic target for anti-metastatic therapy in CRC patients.

## INTRODUCTION

Colorectal cancer (CRC) is one of the most common malignancies in China, and ranks as the third most prevalent cancer and the third leading cause of cancer mortality worldwide [1, 2]. Approximately 50–60% of patients diagnosed with CRC will develop distant metastases. Liver is the most common site and 80–90% of these patients have unresectable metastatic liver disease [3, 4]. Even after undergoing resection of liver metastases, more than 50% of patients will not be cured because of later relapse and development of other distant metastases [5, 6]. Therefore, it is important to find the molecules and pathways through which liver metastases develop in order to further clarify the mechanisms and explore new therapy targets.

MicroRNAs (miRNAs) are a class of small, endogenous, non-coding RNA, which suppress expression of many different gene targets at the post-transcriptional level through sequence-specific interaction with their 3′ untranslated regions (UTRs), leading to translation inhibition or messenger RNA degradation. miRNAs are known to have a function in the process of cancer development and also to play a role in predicting relapse and metastases in CRCs, by regulating the expression of down-stream target genes [7–15]. In these miRNAs, miR-17-5p promotes tumorigenesis and progression by suppressing P130 and activating the Wnt/beta-catenin pathway in CRC [10]. miR-30b inhibits CRC proliferation by inhibiting the expression of KRAS, PIK3CD and BCL2 [9]. Down-regulated miR-224 and its passenger strand promote the growth and metastasis of CRC cells by up-regulating MBD2 [13]. miR-153 promotes invasion and drug resistance by down-regulating ROXO3a [15].

The role of miRNAs in CRC liver metastases has also been investigated in recent years. miR-181a [16] and miR-200c [8] promote liver metastases by inducing epithelial-mesenchymal transition in CRC. miR-192 inhibits liver metastases by down-regulating the expression of Bcl-2, VEGFA and Zeb2 [7]. miR-214 suppresses liver metastases by way of regulation of FGFR1 [17]. However, the actions of miRNAs on CRC liver metastases remain largely unclear and need exploration. This study was performed to investigate the biological function of miRNAs related to CRC liver metastases.

In this study, samples of both the primary CRC and paired liver metastases from more than 50 patients were collected, and a microarray-based strategy was applied to identify the changes in miRNA profile during liver metastases. miR-99b-5p was found to be significantly down-regulated in liver metastases compared with the primary tumor. Its role and mechanisms in tumor metastases were further elucidated.

## RESULTS

### Screening for different expression profiles of miRNAs between the primary CRC and paired liver metastases

**Figure 1:**
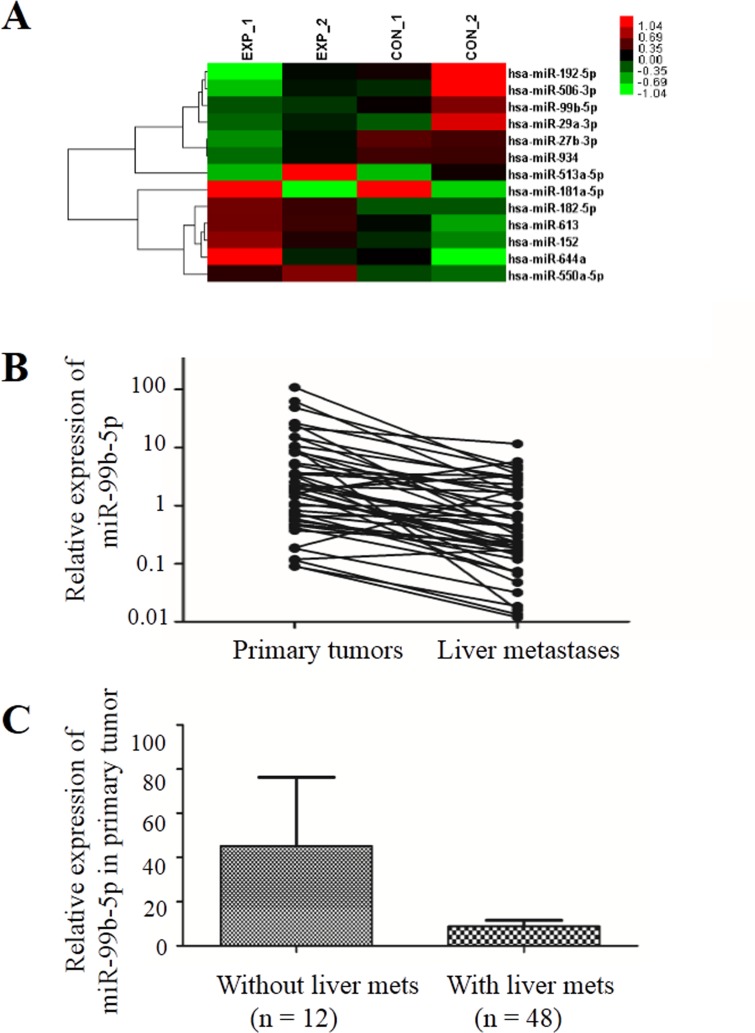
miR-99b-5p was down-regulated in liver metastases compared with the primary colorectal cancer **A**. Affymetrix Human Transcriptome Array 2.0 microarray (Invitrogen, Carlsbad, CA, USA) screened the differential expression of miRNA between primary tumor and liver metastases in two pairs of fresh tissue. **B**. The expression of miR-99b-5p was reduced in liver metastases compared with primary tumor in 48 pairs; *P* = 0.007 (paired t-test). **C**. The expression of miR-99b-5p was reduced in colorectal cancer patients with liver metastases compared with those without liver metastases; *P* = 0.028 (non-paired *t*-test). Abbreviation: met, metastases.

Using microarray analysis, we assessed miRNA expression profiles from two patients' primary CRC fresh frozen tissue samples and their paired liver metastasis tissues to identify miRNAs involved in development of liver metastases. The clinicopathological characteristics of the two patients were well balanced. Seven up-regulated (miR-513a-5p, miR-181a-5p, miR-182-5p, miR-613, miR-152, miR-644a and miR-550a-5p) and six down-regulated (miR-192-5p, miR-506-3p, miR-99b-5p, miR-29a-3p, miR-27b-3p and miR-934) miRNAs were detected in the liver metastases (Figure 1A).

### Expression of miR-99b-5p is significantly reduced in liver metastasis compared with primary tumors and is related to prognosis in CRC patients

In order to confirm the results from microarray analysis, we further determined the miR-99b-5p expression levels in 56 pairs of primary CRC samples and their corresponding liver metastasis tissues (8 pairs of fresh frozen and 48 pairs of paraffin-embedded specimens) by real-time quantitative polymerase chain reaction (PCR). The results showed that expression of miR-99b-5p was down-regulated by more than 6-fold in liver metastasis tissues compared with primary CRCs (*P* = 0.007) (Figure 1B).

**Figure 2:**
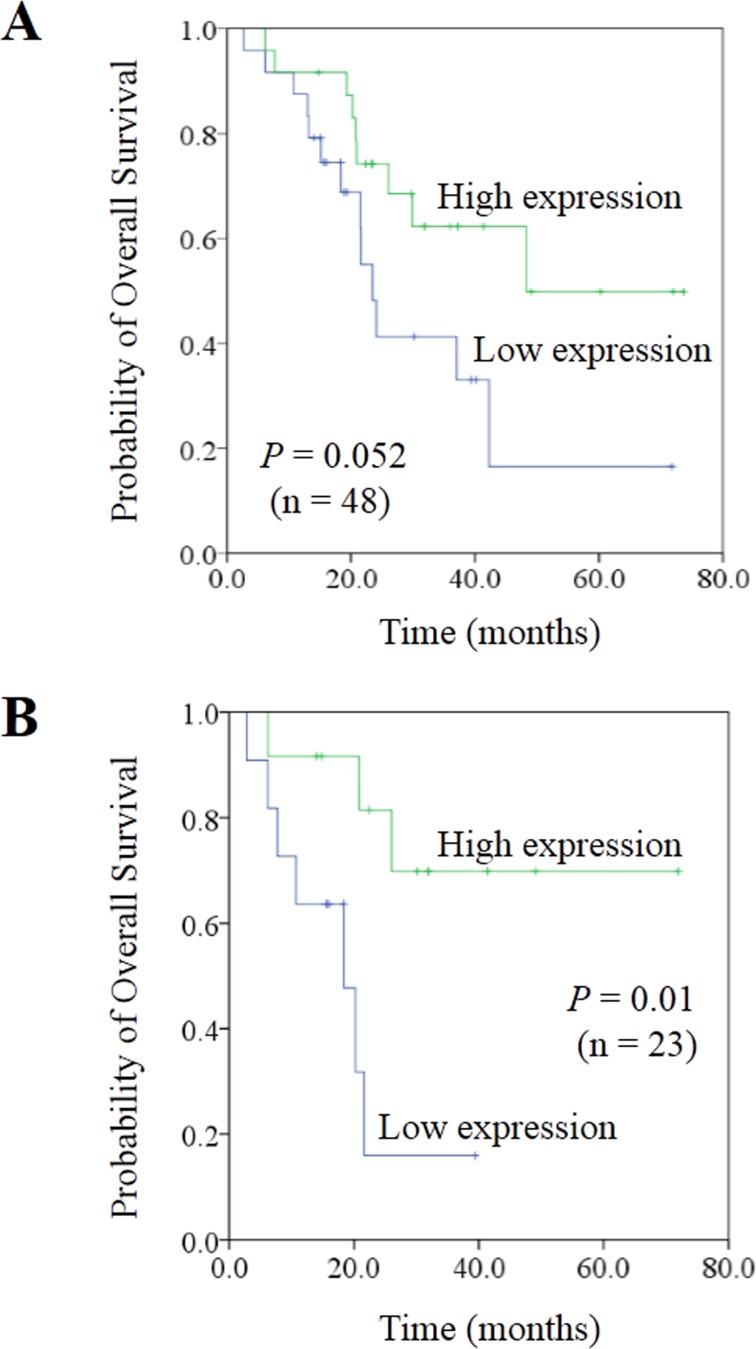
Correlation between expression of miR-99b-5p and prognosis in colorectal cancer liver metastases **A**. In the population of 48 paired colorectal cancer liver metastases patients. **B**. In the population of 23 paired synchronous colorectal cancer liver metastases patients, with liver-limited disease, who had undergone radical resection of both the primary tissue and liver lesions, and had received no chemo- or radiotherapy before the resection.

Moreover, we evaluated the expression of miR-99b-5p in another 12 stage III CRC patients who had not developed liver metastasis 3 years after surgery. These 12 patients had higher miR-99b-5p expression in the primary tumor compared with the 48 CRC patients with liver metastasis (*P* = 0.028) (Figure 1C), suggesting that miR-99b-5p may predict liver metastasis. We evaluated the association between the expression level of miR-99b-5p and patients' survival. Patients with high expression of miR-99b-5p in the primary tumor showed a trend for longer survival time than those with low expression (median overall survival was 48.3 months versus 23.5 months for high expression of miR-99b-5p versus low expression of miR-99b-5p; *P* = 0.052) (Figure 2A). We observed a similar survival trend for the correlation between the miR-99b-5p expression levels in liver metastasis specimens and patient survival (*P* = 0.099).

Considering the influence of previous chemotherapeutic treatment on miRNA expression (Table 1), we excluded patients who had received chemotherapy before obtaining either the primary tumor or liver metastasis tissues. As shown in Figure 2B and Table 2, samples from 23 synchronous CRC patients with liver metastases who were chemotherapy-naïve underwent further analysis of miR-99b-5p expression level and survival. A significant difference was shown, with the median survival time in the miR-99b-5p high-expression group not yet reached, while that in the low-expression group was 18.4 months (*P* = 0.01) (Figure 2B).

**Table 1:** Relationship between miR-99b-5p expression and clinicopathologic parameters in patients with colorectal cancer liver metastases (n = 48)

Characteristic	No.	Percent	miR-99b-5p in primary tumor(mean±SEM)	P value	miR-99b-5p in liver metastases(mean±SEM)	P value
Sex						
Male	29	60.4	9.6387±3.99751	0.719	1.3016±0.29277	0.991
Female	19	39.6	7.5814±3.46567		1.3085±0.62149	
Age (years)						
Median (range)	52 (28-74)					
<60	34	70.8	8.2798±3.61336	0.762	0.8178±0.21728	0.067
≥60	14	29.2	10.1468±3.66777		2.4860±0.81582	
Primary tumor site						
Colon	17	35.4	15.3436±6.99205	0.178	0.9358±0.29816	0.367
Rectum	31	64.6	5.2493±1.70883		1.5064±0.43247	
pT						
2+3	9	18.8	3.8566±1.70966	0.392	1.9291±0.60369	0.321
4	39	81.3	9.9708±3.35167		1.1602±0.33981	
pN						
Negative	12	25.0	12.0637±6.05071	0.503	1.3266±0.45696	0.966
Positive	36	75.0	7.7446±3.10294		1.2969±0.37175	
Perineural invasion						
Positive	16	37.2	3.2499±0.96070	0.043*	0.4867±0.21087	0.019*
Negative	27	62.8	13.4533±4.71018		1.7473±0.46550	
NK	5					
Vascular invasion						
Positive	16	37.2	4.1719±1.63814	0.088	0.6909±0.26904	0.153
Negative	27	62.8	12.9069±4.68848		1.6263±0.46622	
NK	5					
Differentiation						
Poor/moderate-poor	12	25.0	3.6132±1.08278	0.074	0.8460±0.29063	0.382
Moderate/high-moderate	36	75.0	10.5614±3.62343		1.4571±0.38544	
Occurrence of liver metastatses						
Synchronous	43	89.6	9.0836±3.04764	0.786	1.0929±0.22571	0.397
Metachronous	5	10.4	6.5948±4.06253		3.1231±2.13569	
Chemotherapy before the operation of primary tumor						
Yes	8	16.7	1.7486±1.04913	0.255	0.2004±0.06598	0.001*
No	40	83.3	10.2395±3.26106		1.5251±0.34876	
Chemotherapy before the operation of liver metastasis						
Yes	22	45.8	9.0397±3.49834	0.944	0.7426±0.25405	0.07*
No	26	54.2	8.6422±4.20185		1.7796±0.49469	
Combined with other metastases						
Yes	7	14.6	4.0009±1.47293	0.475	0.8070±0.39800	0.498
No	41	85.4	9.6479±3.20540		1.3893±0.34306	

Abbreviations: NK, not known; pN, pathologic node; pT, pathologic tumor; SEM, standard error of the mean.

* Denotes significance.

**Table 2:** Relationship between miR-99b-5p expression and clinicopathologic parameters in chemotherapy-naïve patients with synchronous colorectal cancer liver metastases (n = 23)

Characteristic	No.	Percent	miR-99b-5p in primary tumor(mean±SEM)	P value	miR-99b-5p in liver metastases(mean±SEM)	P value
Sex						
Male	13	56.5	11.8715±8.14648	0.455	1.7069±0.49799	0.364
Female	10	43.5	4.5740±2.45382		1.0710±0.43560	
Age (years)						
Median (range)	52 (28-72)					
<60	16	69.6	9.0431±6.68341	0.915	1.3413±0.41877	0.700
≥60	7	30.4	7.9114±3.34287		1.6343±0.60836	
Primary tumor site						
Colon	5	21.7	27.89±20.68175	0.302	1.8260±0.85487	0.551
Rectum	18	78.3	3.3678±0.97034		1.3206±0.37239	
pT						
2+3	5	21.7	4.644±2.76588	0.660	2.2700±0.97958	0.198
4	18	78.3	9.8250±5.97868		1.1972±0.33507	
pN						
Negative	4	17.4	1.8875±0.64400	0.519	1.0925±0.73401	0.658
Positive	19	82.6	10.1326±5.66261		1.5016±0.38629	
Perineural invasion						
Positive	8	34.8	2.1937±1.25798	0.24	0.56±0.37358	0.037*
Negative	11	47.8	15.9673±9.52915		1.9091±0.43051	
NK	4	17.4				
Vascular invasion						
Positive	5	21.7	3.1360±1.94324	0.474	0.7680±0.59202	0.309
Negative	14	60.9	12.6793±7.61171		1.5457±0.38797	
NK	4	17.4				
Differentiation						
Poor/moderate-poor	7	30.4	3.6797±1.59738	0.493	1.1413±0.46560	0.583
Moderate/high-moderate	16	69.6	10.8951±6.71938		1.5578±0.44751	
Combined with other metastases						
Yes	4	17.4	4.4200±2.15275	0.686	1.1900±0.66402	0.753
No	19	82.6	9.5995±5.68460		1.4811±0.39173	

Abbreviations: NK, not known; pN, pathologic node; pT, pathologic tumor; SEM, standard error of the mean.

* Denotes significance.

### Expression of miR-99b-5p suppresses metastasis in CRC cells *in vitro*

**Figure 3:**
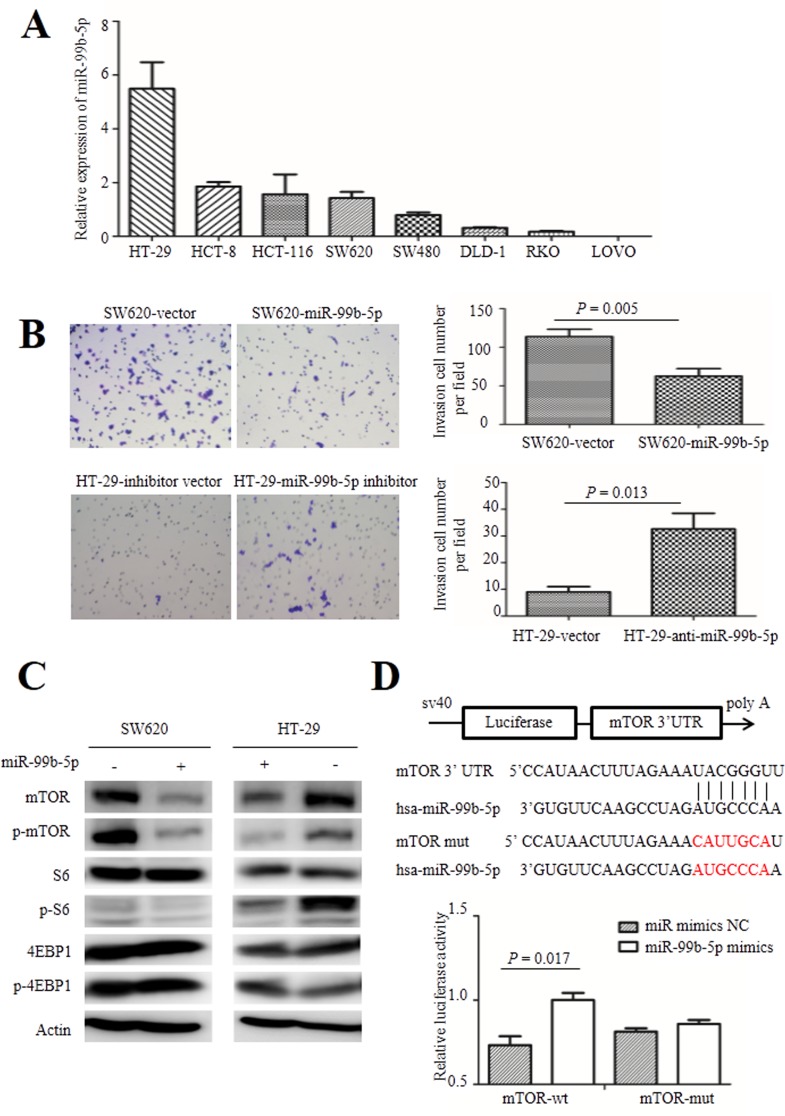
Role of miR-99b-5p in colorectal cancer cells **A**. The expression level of miR-99b-5p in human colorectal cancer cells. **B**. The migration ability of colorectal cancer cells were analyzed after transfection with miR-99b-5p (original magnification, x 200). The experiment was repeated three times and the error bars represent ± standard deviation. **C**. Expression of mTOR, phospho-mTOR (Ser2448), S6, phospho-S6 (Ser235/236), 4EBP1 and phospho-4EBP1 (Thr37/46) were analyzed by Western blot. **D**. WT and Mut 3′ UTRs of mTOR, indicating the interaction sites between miR-99b-5p and 3′ UTR of mTOR. Dual luciferase assay of HEK-293T cells cotransfected with the firefly luciferase constructs containing the mTOR-wt or mTOR-mut 3′ UTR and miR-99b-5p mimics or miR-99b-5p mimics NC. Data are shown as mean ± standard deviation from at least three independent experiments. Abbreviations: Mut, mutant-type; NC, negative control; UTR, untranslated region; WT, wild-type.

Based on the observations of miR-99b-5p in patients with CRC, overexpression of miR-99b-5p in CRC cells can exert inhibitory effects on cell invasion and metastasis. In order to examine the effect of miR-99b-5p on CRC cell migration, we first tested the basic expression level of several CRC cells by real-time PCR. As shown in Figure 3A, low expression of miR-99b-5p was observed in LOVO, SW620, SW480, RKO and DLD-1 cells, while relatively higher expression was detected in HT-29 cells.

Next, we transfected the miR-99b-5p mimics into SW620 cells and confirmed the successful overexpression of miR-99b-5p by real-time PCR. We found that overexpression of miR-99b-5p significantly suppressed the migratory ability of SW620 cells (*P* = 0.005) (Figure 3B). As a contrast, we transiently transfected miR-99b-5p inhibitors into HT-29 cells, which had relatively high endogenous miR-99b-5p expression among CRC cell lines and down-regulation of miR-99b-5p promoted CRC cell migration (*P* = 0.013) (Figure 3B). The proliferation ability of colon cells were not influenced by the transfection of miR-99b-5p mimics or inhibitors, as was shown in Supplemental Figure 1.

### miR-99b-5p inhibits expression of mTOR by directly targeting its 3′ UTR *in vitro*

To explore the molecular mechanism of miR-99b-5p in CRC liver metastasis, we used several databases including TargetScan, miRBase and PicTar bioinformation to search for putative protein-coding gene targets of miR-99b-5p, especially for those that have the abilities to promote tumor cell invasion and metastasis. As a result, mTOR was selected as a candidate gene. mTOR was reported to inhibit CRC tumor formation and the mTOR pathway also plays a crucial role in cancer biology, including the upstream activator Akt and down-stream molecules S6, 4EBP1 and their phosphorylation proteins [18]. Western blot analysis showed that enhanced expression of miR-99b-5p triggered a silencing effect on mTOR protein expression and its phosphorylation. After transfection with the miR-99b-5p inhibitor in HT-29 cells, expression of mTOR and its phosphorylation products were increased. Furthermore, the down-stream S6 molecule exhibited a similar regulatory trend, while expression of 4EBP1 and p-4EBP1 showed no obvious changes (Figure 3C).

To test whether mTOR is a direct target of miR-99b-5p, a series of 3′ UTR fragments of mTOR, including the binding site and its corresponding mutated counterpart, were directly fused down-stream of the firefly luciferase gene. As shown in Figure 3D, miR-99b-5p could decrease the relative luciferase activity of the mTOR 3′ UTR construct (*P* = 0.017) whereas, in the counterpart with the mutated site, the luciferase activity was not significantly changed (*P* = 0.205), indicating that miR-99b-5p down-regulates mTOR expression by directly targeting its 3′ UTR (Figure 3D).

To confirm that mTOR is a functional target of miR-99b-5p, we further explored whether inhibition of mTOR could mimic the effect of ectopic expression of miR-99b-5p. In SW620 cells, knockdown of mTOR suppressed cell migration ability (*P* = 0.0021), as was shown in Supplemental Figure 2. The restoration experiment of mTOR in HT-29 cells should have been done, but it did not complete because of the technical difficulty in transfecting the plasmid containing mTOR, which is too large (CCDS nucleotide sequence of mTOR: 7.65kbp).

### mTOR is a critical factor in CRC metastasis, and up-regulation of mTOR is inversely correlated with miR-99b-5p expression in CRC

**Figure 4:**
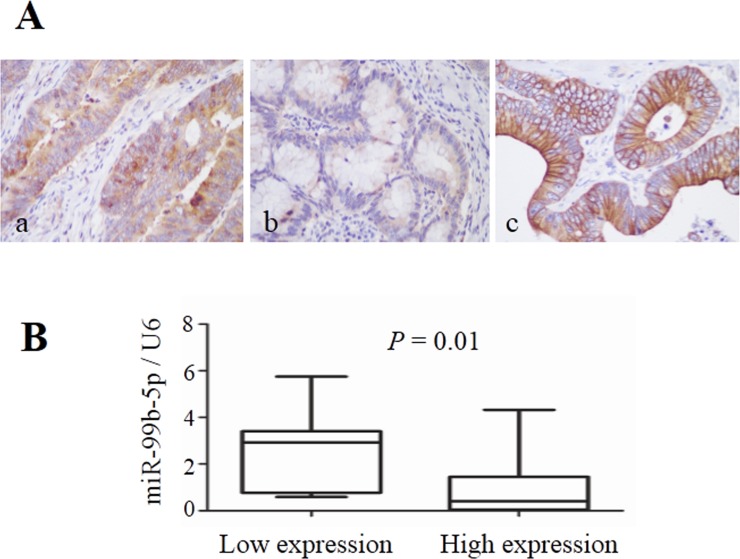
Expression of mTOR in colorectal cancer liver metastases and its relationship with miR-99b-5p **A**. mTOR expression in colorectal cancer liver metastases (original magnification, x 400): (a) high expression in colon cancer tissue, (b) low expression in colon cancer tissue and (c) high expression in liver metastatic tissue. **B**. The expression level of miR-99b-5p in mTOR-high expression patients was lower than that in mTOR-low expression patients; *P* = 0.01.

**Figure 5:**
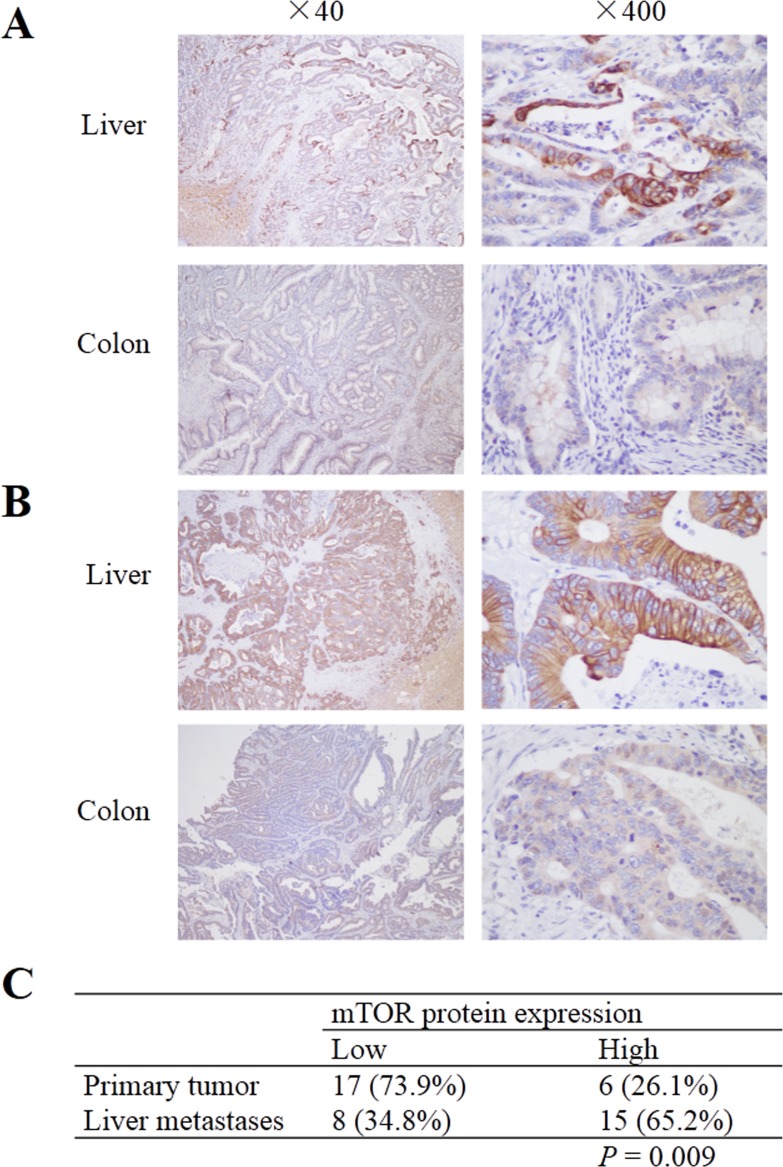
mTOR expression in primary colorectal cancer and corresponding liver metastases **A** and **B**. Samples from two patients who showed low expression of mTOR in primary tumor, but high expression in liver metastatic specimens. **C**. mTOR expression was higher in liver metastases than in the primary tumor; *P* = 0.009.

**Figure 6:**
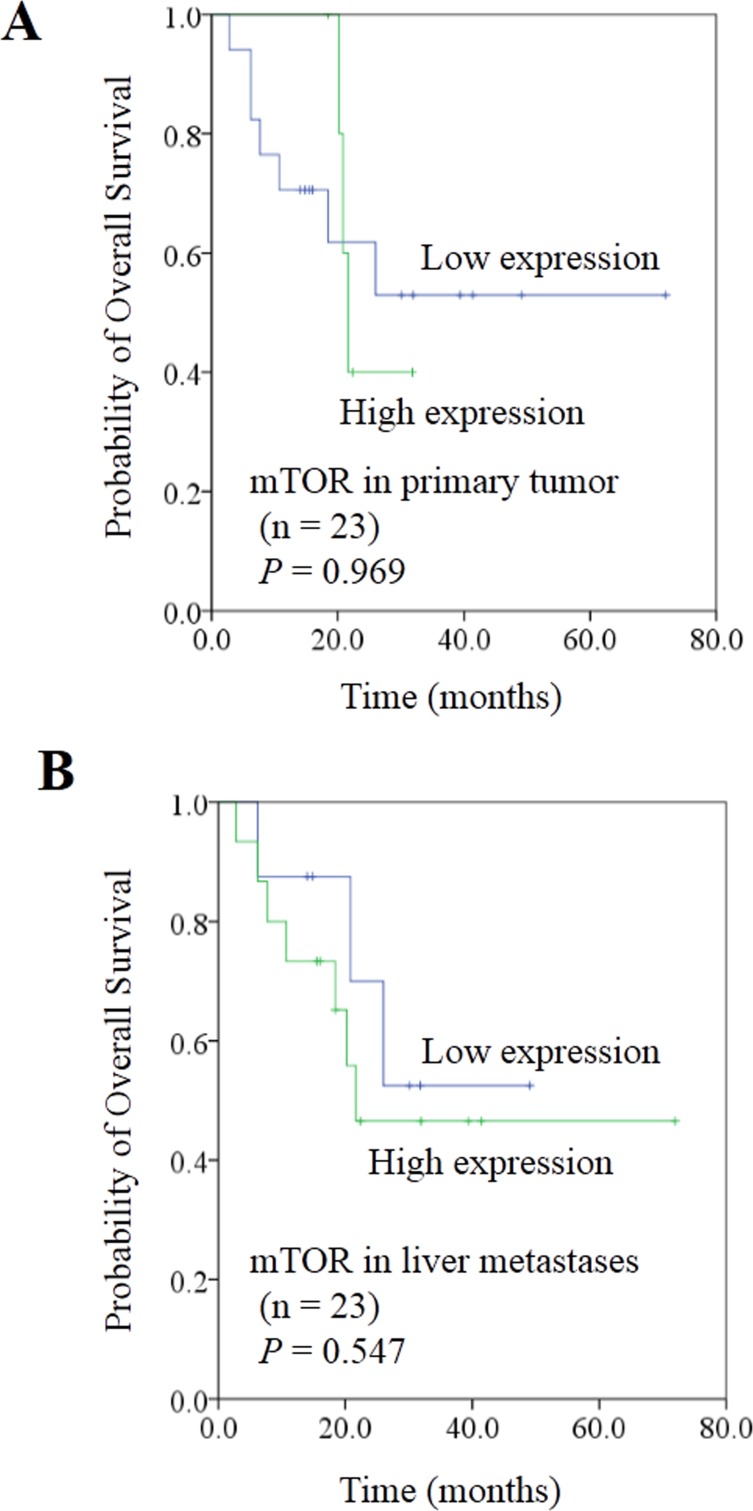
Correlation between expression of mTOR and prognosis in colorectal cancer liver metastases **A**. mTOR expression in primary tumor did not show a predictive effect on overall survival. **B**. Patients with high mTOR expression in liver metastasis showed a trend for worse overall survival than those with low mTOR expression; P value did not reach significance.

To evaluate the correlation between mTOR and miR-99b-5p, the protein expression of mTOR and its down-stream pathway genes were examined by immunohistochemistry. Our results showed that the expression of miR-99b-5p was negatively associated with mTOR expression level in the 23 CRC patients with liver metastases (*P* = 0.01) (Figure 4, Table 3). We also found that the down-regulated expression of mTOR in the primary tumor tissues was comparable with that of the corresponding liver metastases (Figure 5). However, correlation of mTOR in the primary tumor and overall survival was not observed. Higher expression of mTOR in liver metastases showed a poorer trend for survival, although a *P* value was not reached (Figure 6).

**Table 3:** Relationship between miR-99b-5p expression and mTOR/p-mTOR/S6/p-S6 in patients with colorectal cancer liver metastases (n = 23)

		No.	miR-99b-5p in primary tumor	P value	No.	miR-99b-5p in liver metastases	P value
mTOR	Low expression	17	8.4106±6.27113	0.921	8	2.5763±0.62146	0.01
	High expression	6	9.5150±4.05587		15	0.8193±0.31105	
p-mTOR	Low expression	15	2.2820±0.59777	0.197	13	1.5123±0.41732	0.790
	High expression	8	20.73±12.91816		10	1.3240±0.58387	
S6	Low expression	9	2.6011±0.80422	0.31	3	2.1967±1.13702	0.393
	High expression	14	12.6186±7.63147		20	1.3155±0.35640	
p-S6	Low expression	19	4.6342±1.50482	0.448	14	1.0857±0.34128	0.096
	High expression	4	28.005±26.7964		9	1.9667±0.67290	

## DISCUSSION

Recent studies have highlighted the role of miRNAs in a broad range of developmental processes of carcinogenesis and progression [19, 20]. Comparative genomics have been widely used to find the novel target genes by using the high-throughput technologies [21, 22]. Similar methods have been applied to gain better insight into genomic changes involved in cancer metastasis [23]. In this study, we used a microarray approach to identify the key regulatory miRNAs responsible for liver metastasis by comparing the expression differences of miRNAs between the primary cancer and paired liver metastatic lesions. By exploring the patients' samples, seven up-regulated (miR-513a-5p, miR-181a-5p, miR-182-5p, miR-613, miR-152, miR-644a and miR-550a-5p) and six down-regulated (miR-192-5p, miR-506-3p, miR-99b-5p, miR-29a-3p, miR-27b-3p and miR-934) miRNAs were detected. Among them, miR-99b-5p was found to play an inhibitory role in CRC metastasis and was associated with favorable survival in CRC patients with liver metastasis. To our knowledge, this is the first study to investigate the role of miR-99b-5p in CRC liver metastasis. We found that miR-99b-5p was expressed significantly less in liver metastasis than in the corresponding primary tumor. Moreover, miR-99b-5p expression was reduced in CRC patients with liver metastases compared with those without liver metastases. Previously, miR-99b has been found to be deregulated in several tumors such as pancreatic cancer, esophageal cancer and endometrial cancer [24–27]. However, the biological role of miR-99b-5p in CRC remains unclear. Importantly, miR-99b-5p was associated with a survival benefit in CRC patients with liver metastases, suggesting that miR-99b-5p may serve as a predictive factor for metastasis and prognosis of CRC patients.

Given that miR-99b-5p is down-regulated in CRC patients with liver metastasis, we speculated that miR-99b-5p may inhibit metastasis in CRC. Functional assay of miR-99b-5p in tumor cell lines was done to support the findings from the clinical samples. Our results demonstrated that overexpression of miR-99b-5p could inhibit migration of CRC cells *in vitro*, and vice versa. Interestingly, previous studies have reported that miR-99b could be tumor suppressive in certain cancer types such as prostatic cancer [28], non-small cell lung cancer [29] and cervical cancer [30]. These results highlight the role miR-99b-5p in CRC liver metastasis, suggesting that targeting miR-99b-5p could be a promising therapeutic measure in CRC patients.

It is well known that miRNAs participate in various physiological and pathological processes by directly regulating target gene expression. To explore the underlying mechanism of miR-99b-5p function in CRC, we searched for direct target genes regulated by miR-99b-5p by bioinformation analysis. We detected several putative targets, including mTOR, HOXA1, TRIB2 and FGFR3. Further investigation showed that mTOR was a direct functional target of miR-99b-5p in CRC. First, a silencing effect of mTOR expression and phosphorylation was observed after enhancing miR-99b-5p in CRC cells. Second, ectopic miR-99b-5p expression markedly reduced the activity of a luciferase reporter containing the 3′ UTR sequence of mTOR. Third, mTOR overexpression was frequently observed in liver metastases compared with the primary tumor, and an inverse correlation was found between the expression levels of miR-99b-5p and mTOR in CRC patients with liver metastasis. Interestingly, a recent report shows that the miR-99b–mTOR axis is also present in dermal wound healing [31] and in irradiation resistance in pancreatic cancer [32], which are in accordance with our results in CRC.

mTOR is a serine/threonine protein kinase, which is a key effector in the PI3K-Akt-mTOR pathway [33]. As a proto-oncogene, mTOR promotes cell growth and progression as well as activates human tumorigenesis [34]. The mTOR pathway has been reported to be involved in bladder cancer [35], metastatic kidney cancer [36], hepatocellular carcinoma [37] and glioma [38]. More importantly, targeting mTOR with small-molecule inhibitors has exhibited antitumor activity for malignant tumor in clinical trials. The allosteric inhibitors of mTOR, everolimus and temsirolimus, have shown promising clinical benefit in advanced renal cell carcinoma [39]. A phase I study found that another novel inhibitor of mTOR, ridaforolimus, also exhibited promising efficacy in advanced solid tumors [40]. Besides the use of mTOR inhibitors in treating cancer, rapamycin and other rapalogs were also reported to have indirectly cancer-preventive effects in human by slowing aging [41]. In the present study, we found that mTOR was overexpressed in liver metastatic lesions and patients with mTOR-overexpressed disease showed a trend for poor survival, although the P value was not reached due to the small sample size. Given that the mTOR pathway has been demonstrated to participate in regulating epithelial-mesenchymal transition, motilityand metastasis of CRC cells in previous studies [42, 43], our results showed the role of the miR-99b-5p–mTOR axis in CRC metastases in a more comprehensive way, both in cell lines and clinical samples. These data suggest an important role for mTOR in the development of CRC liver metastasis, and the miR-99b-5p–mTOR axis may shed more light on finding new strategies for metastatic CRC treatment.

In summary, our results provide new clues for the role of miR-99b-5p in CRC liver metastatic clinical samples and cell lines. We demonstrated that miR-99b-5p functions as a tumor suppressing miRNA in metastatic CRC, and that its suppressive effects are mediated chiefly by suppressing mTOR expression. These findings are essential for understanding the molecular mechanism of liver metastasis in CRC patients and providing a novel therapeutic target for treatment. miR-99b-5p may be a potential prognostic factor, and the miR-99b-5p–mTOR axis could be further explored as a therapeutic target for anti-metastatic therapy in CRC patients with liver metastases.

## MATERIALS AND METHODS

### Human tissue specimens

Human CRC and liver metastatic tissues were collected at the time of surgical resection in the Department of Colorectal Cancer, Fudan University Shanghai Cancer Center, Shanghai, China, from 2004 to 2012. The tumor samples were pathologically confirmed and evaluated for tumor content by a pathologist (median tumor content in the samples was 80–90%). Both the fresh frozen specimens and formalin-fixed, paraffin-embedded specimens were stored in the tissue bank of the hospital. The tumor tissues were sliced into 10 μm sections using a cryostat microtome, aliquoted into 1.5 mL Micro tubes (Sarstedt, Nümbrecht, Germany) and stored at −80°C. Signed informed consent was obtained from all patients and the study was approved by the Clinical Research Ethics Committee of Fudan University Shanghai Cancer Center.

### Cell lines and reagents

The human colon carcinoma HT-29, HCT-8, HCT-116, SW480, SW620, DLD-1, RKO and LOVO cell lines were cultured in Roswell Park Memorial Institute (RPMI) 1640 medium with 10% fetal bovine serum (FBS). miR-99b-5p duplex mimics and negative control (NC), and inhibitors and NC were purchased from GenePharma (Shanghai, China).

Sequences for RNA oligo were as follows:

miR-99b-5p mimic: 5′ CACCCGUAGAACCGACCUUGCG 3′; 5′ CAAGGUCGGUUCUACGGGUGUU 3′

miR-99b-5p inhibitor: 5′ CGCAAGGUCGGUUCUACGGGUG 3′

miRNA negative control: 5′ UUCUCCGAACGUGUCACGUTT 3′; 5′ ACGUGACACGUUCGGAGAATT 3′

miRNA inhibitor negative control: 5′ CAGUACUUUUGUGUAGUACAA 3′.

RPMI-1640, FBS and serum-free opti-MEM^TM^ were obtained from Gibco (Carlsbad, CA, USA). X-tremeGENE siRNA Transfection Reagent was purchased from Roche (Shanghai, China). Goat anti-rabbit and mouse horseradish peroxidase-conjugated immunoglobulin G were purchased from Santa Cruz Biotechnology, Inc (Santa Cruz, CA, USA). All other phospho- and non-phospho- antibodies were purchased form Cell Signaling Technology (Denver, MA, USA). The enhanced chemiluminescence Western blot reagent kit was purchased from Pierce (Rockford, IL, USA).

### Transfection

HT-29 and SW620 cells were seeded in 6-well plates (2 × 10^5^/well) and starved for 3 hours. Cells were transfected with miR-99b-5p precursor or inhibitor, opti-MEM and X-tremeGENE siRNA Transfection Reagent according to the manufacturer's instructions, and cultured in RPMI 1640 medium with 10% FBS. Cells were used for migration assay after 24 hrs of transfection. Cells were harvested and lysed for Western blot assay after 48 hrs of transfection.

The siRNA against mTOR was synthesized by Transheep Bio-Tech Co., Ltd. The sequence of siRNA targeting mTOR was as follows: 5′ GGCACAAUGCAGCCAACAATT 3′. Transfection of oligonucleotides construction was performed using the Lipofectamine 3000 reagent (Life Technologies, Carlsbad, CA, USA) according to the manufacturer's instructions.

### RNA isolation and quantitative real-time PCR

Total RNA was isolated from cells using TRIzol® reagent (Invitrogen, Carlsbad, CA, USA) and formalin-fixed paraffin slices using RecoverAll^TM^ Total Nucleic Acid Isolation Kit (AM1975, Ambion®; Life Technologies, Carlsbad, CA, USA), according to the protocol. Quantitative PCR was performed using miRNA Reverse Transcription Kit (Applied Biosystems, Carlsbad, CA, USA), TaqMan® PreAmp Pools (Applied Biosystems) and LNA^TM^ miRNA Quantitative Fluorescence Detection Kit (HaoQin, Shanghai, China) with a Universal ProbeLibrary (Roche, Basel, Switzerland):

miR-99b-5p: MIMAT0000689; CACCCGUAGAACCGACCUUGCG;

U6 was used as an endogenous reference gene and the data were analyzed using the 2^−dCt^ method. The experiment was repeated three times independently.

### Migration assay

2 × 10^5^ cells of SW620 cells in 300 μL 5% FBS medium were loaded into Transwell upper inserts with 8 μm pore polycarbonate filters (BD Falcon^TM^ Cell Culture Inserts, Sterile; BD Biosciences, Franklin Lakes, NJ, USA) and 500 μL 30% FBS medium were loaded into the lower chamber. After 24 hrs incubation, the invaded cells on the bottom side of the membrane were fixed and stained with 0.005% crystal violet. The number of invaded cells was counted in five randomly chosen areas (200 × magnification) and summarized. A similar method was performed for HT-29 cells except that 5 × 10^5^ cells were loaded into every insert.

### Cell proliferation assay

Assessment of cell viability was performed as follows. Cells were seeded at 5,000 cells per well in 96-well plates and incubated for 96 hours. Cell viability was determined at 24, 48, 72 and 96 hours using the CCK-8 according to the manufacturer's instructions.

### Luciferase assays

HEK-293T cells were seeded in 96-well plates at 3 × 10^4^ cells per well the day before transfection. A mixture of 200 ng indicated psiCHECK2-3′ UTR, 5 pmol miR-99b-5p mimics was transfected into HEK-293T cells with LipoFiter^TM^ (Hanbio, Shanghai, China) in each well. Thirty-six hrs later, Firefly and Renilla luciferase activities were measured with a Dual-Luciferase® Reporter System (Promega, Madison, WI, USA). The Firefly luciferase activities were used as an internal control for transfection efficiency.

### Western blot assay

Cells were lysed in radioimmunoprecipitation assay buffer supplemented with complete protease inhibitor cocktail (Roche, Basel, Switzerland). Protein concentrations were determined using the BCA protein assay kit (Biyotime, Shanghai, China). Antibodies against mTOR, p-mTOR, S6, p-S6, 4EBP1 and p-4EBP1 were purchased from Cell Signaling Technology (Cambridge, MA, USA). Antibody against β-actin was purchased from Jackson Laboratories (Bar Harbor, ME, USA). Blots were probed with indicated primary antibodies, then incubated with the horseradish peroxidase-conjugated secondary antibody and detected by enhanced chemiluminescence reagent.

### Immunohistochemistry

Four μm thick paraffin sections were stained for mTOR, p-mTOR, S6 and p-S6 expression. Slices were deparaffinized and hydrated. Antigen retrieval was achieved by pressure cooking in 10 mM sodium citrate buffer, pH 6.0 for 10 mins, and incubating with primary antibody overnight (1:100 for mTOR, p-mTOR and S6, 1:400 for p-S6) at 4°C. Detection took place by GT Vision^TM^ III Detection System/Mo Rb (GeneTech, Shanghai, China) and colorimetric detection with 3,3′-diaminobenzidine. Afterwards, the slides were dehydrated and mounted with coverslips.

### Statistical analysis

Data are shown as mean ± standard error of the mean unless otherwise noted. Two-sided paired student's t-test was used to compare the different expression between the primary tumor and liver metastases. Association between miRNA and clinicopathologic characteristics/gene expression were explored using the independent-sample t-test and one factor analysis of variance as appropriate. Survival was estimated using the Kaplan-Meier method and compared using the log-rank test. All statistical analyses were performed using the Statistical Package for the Social Sciences version 18.0 (SPSS, Inc, Chicago, IL, USA) and the results were considered statistically significant at *P* < 0.05.

## SUPPLEMENTAL MATERIAL FIGURES


