# Supplementary material for: miRNA-99b-5p suppresses liver metastasis of colorectal cancer by down-regulating mTOR
Source: Oncotarget. 2015 Jun 10;6(27):24448–62. doi: 10.18632/oncotarget.4423 (PMC4695197; doi:10.18632/oncotarget.4423)
Supplement: Supplementary file 1 [file oncotarget-06-24448-s001.pdf]

miRNA-99b-5p suppresses liver metastasis of colorectal cancer by down-regulating mTOR

Supplemental Material

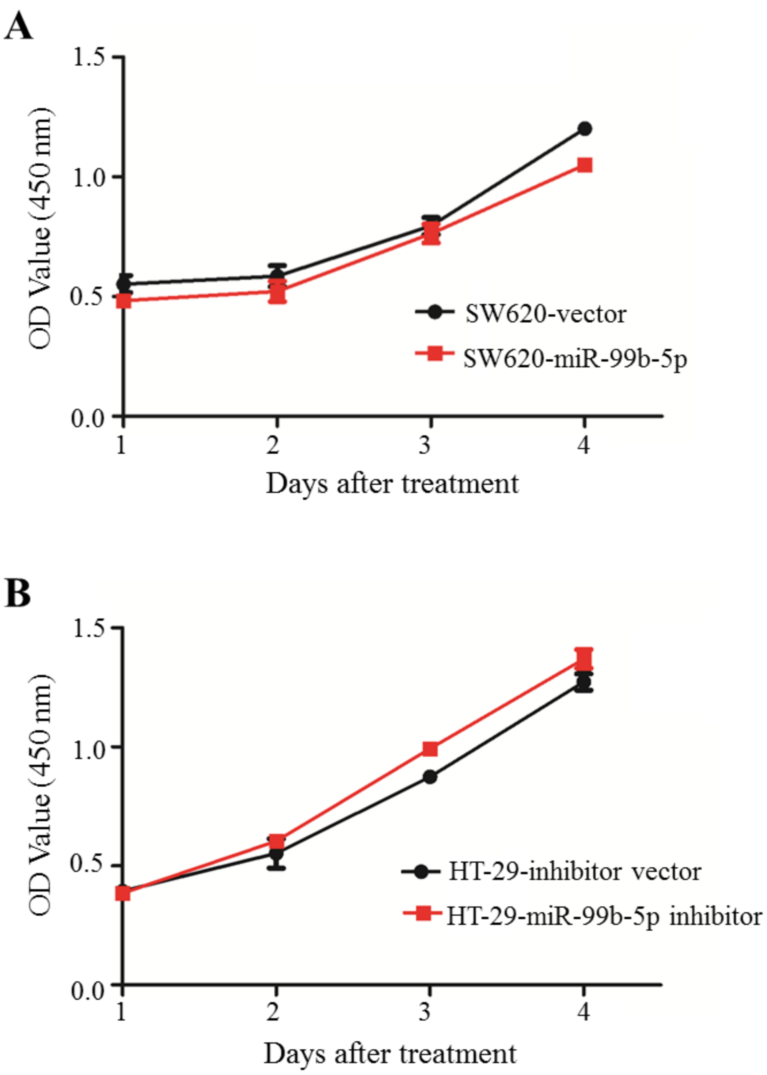

Figure S1: miR-99b-5p did not influence cell proliferation ability of HT-29 and SW680 cells.

SW680 (A) and HT-29 (B) cells were transfected with miR-99b-5p mimic and miR-99b-5p inhibitor, respectively. CCK-8 assay was performed to test cell proliferation after transfection on day 1, 2, 3 and 4. Data are shown as mean ± standard deviation (n=6).

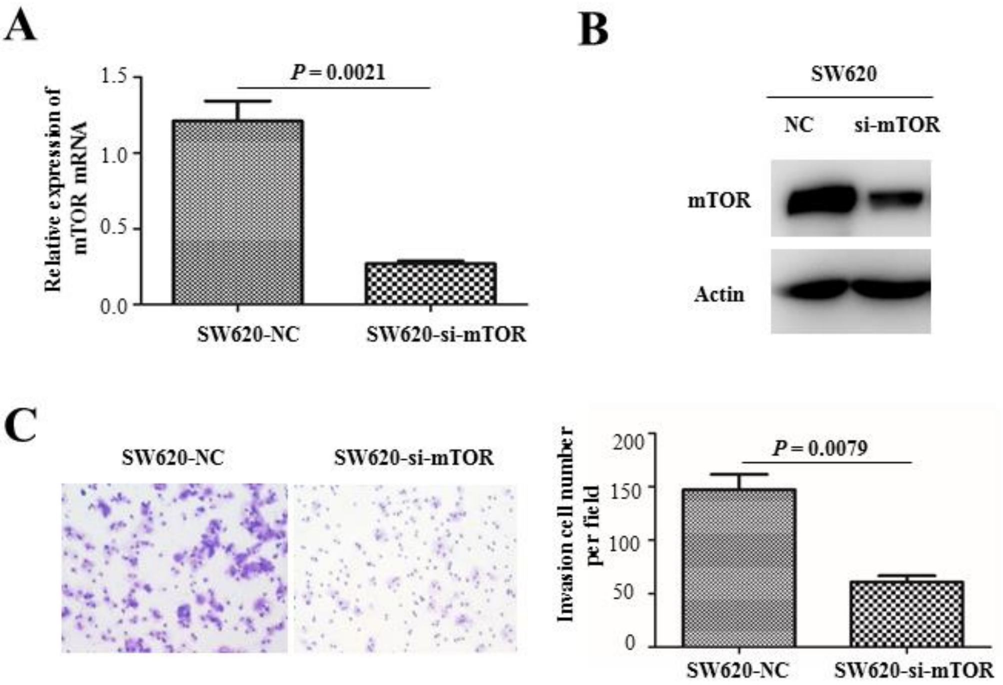

Figure S2: mTOR is involved in miR-99b-5p-induced suppression of colorectal cancer cell migration.

Migration assay was carried out in SW620 cells after transfection with negative control or si-mTOR (original magnification, x 200). The experiment was repeated three times and the error bars represent ± standard deviation. Silencing of mTOR was confirmed by real-time PCR (A) and Western blot (B) in SW620 cells after transfection with specific si-mTOR.
